# Commentary to Skudlik et al. (2023): why a scoping review and why only Germany?

**DOI:** 10.1186/s12912-024-02079-5

**Published:** 2024-06-17

**Authors:** Bich-Lien Nguyen

**Affiliations:** 1 Faculty of Nursing, Université de Montréal, C.P. 6128, succursale Centre-ville, Montréal, QC H3C 3J7 Canada; 2 School of Nursing, Université de Sherbrooke, Campus de Longueuil, 150 Place Charles-Le Moyne. C P. 200, Longueuil, Québec J4K 0A8 Canada

**Keywords:** Commentary, Scoping review, Admission to nursing homes

## Abstract

This letter to the editor is a commentary on the scoping review by Skudlik et al. (2023) on the relocation of older people to nursing homes in Germany. In this commentary, we question certain methodological decisions that, in our view, particularly affect transferability of the results and give a partial picture of the phenomena studied by limiting the inclusion to German studies. We also have questions about the choice of knowledge synthesis method and why the concept of “nursing home” was not defined. We hope that this letter will open a constructive scientific discussion on an important topic that is understudied as the world’s population ages.

## To the Editorial Board, 

We read the recently published scoping review by Skudlik, Hirt [1] with great interest, as it reports on an important topic, the challenges and care strategies when older people are relocated to nursing homes in Germany. We appreciated the holistic perspective of this scoping review, which included patients, family caregivers and health care providers. As the worldwide population is aging, there is a need to restructure healthcare in societies. However, although it presents interesting results on relevant research questions, it also raises questions about some methodological choices. Our main concern is the decision to include only German studies in the scoping review, which severely limits the use of the results in other contexts. We also question the decision to use a scoping review as the preferred method to reach the aim of this review. We also wonder why the concept "nursing home" is not defined. With this commentary, we hope to engage in a constructive scientific discussion of these choices.

Why only Germany? We found the decision to include only German studies very restrictive and methodologically questionable, especially when conducting a scoping review. As its name suggests, a scoping review is exploratory and should be broad in scope to map scientific knowledge. Therefore, the focus should be on all relevant knowledge about a phenomenon. So why not extend the scoping review to other countries with similar health care systems in Europe or even worldwide instead of focusing only on one country? This would have broadened the scope of this literature review. By limiting the selection of studies to Germany, we only get a partial picture of the literature, which may only apply to the German context. This seems to be less useful for the international scientific community.

The rationale provided for this methodological choice is that contextual and country-specific knowledge is needed to improve transitions to nursing homes in Germany and to overcome implementation challenges of interventions available from other countries’ literature. Available scientific knowledge about a similar context in another country could still be useful and transferable to the German context. While the justification provided in Skudlik’s article seems logical, we believe that it is not entirely congruent with the purpose of a scoping review. Even if a justification is given for conducting this type of review using the JBI manual [2], the fact remains that it is somewhat contradictory to limit such a review to a single country. For example, wouldn't it have been more appropriate to carry out a systematic review of intervention effectiveness or a mixed review instead?

Moreover, the article concludes that most German studies on relocation to nursing homes focused on caregivers and health professionals and not much on patients. This finding could have been contrasted with peer-reviewed studies from the international literature, as it has been discussed by multiple articles in recent international studies [3–7]. This is an additional argument in relation to the questionable decision to have limited the review to Germany. As a result, we have only a partial picture of the phenomenon under study which affects the transferability of the results [8, 9]. Although the description of the healthcare system in Germany helped the reader to understand the target setting, being from another country, we found it insufficient to ensure transferability to other contexts [9].

Why a scoping review? As already mentioned, we also question the use of a scoping review instead of a systematic review or a mixed review. As described by Tricco et al., (2016) [10], cited in the JBI manual for evidence synthesis (Peters et al., (2020), p.408 [2])), the three main reasons for conducting a scoping review are to: “explore the breadth or extent of the literature, map and summarize the evidence, and inform future research”. To the best of our knowledge, the justification for this method is not precisely related to any of these reasons. The article mentions implementation challenges of existing interventions as a justification, which is not usually the goal of a scoping review [2]. According to Munn et al., (2018) [11], quoted in Peters et al., (2020) [2] on page 408, if authors want to use the results of their review to develop a clinical guideline, to answer a clinical question, or to provide evidence to inform practice or policy, a systematic review would be most appropriate.

As the article by Fontaine et al. (2022) indicates [8], the first step in deciding why to conduct a knowledge synthesis, such as a scoping review, is to acknowledge if it is required and for what scientific reasons. Hence, a stronger justification of the identified knowledge gap should have been presented in the light of current scientific knowledge. It is not clear to us that there was a lack of knowledge in the international scientific literature (not just specific to the German context) to justify carrying out a scoping review on the subject.

So, it seems questionable to conduct a scoping review when the focus is only on one country, and then offer a narrow scope that does not take into account the state of the science on the topic. This limits the usefulness of the results to the international scientific community which would have benefited from a broader knowledge synthesis on such an important topic. If this decision was influenced by limited time, resources, or lack of planning, this should be mentioned [8].

Why not define the concept of nursing home? Although the eligibility criteria of studies were easily found in Skudlik et al. (2023)'s article based on the Population-Concept-Context (PCC) format recommended by the JBI manual [2], we noticed that the central concept of “nursing home” was not defined, limiting a clear understanding of the scope of the review and how the results could be transferable. The concept of a nursing home can, from one country to another, represent an institution with variable services and populations with different clinical profiles and independence status. As this concept has different meanings from one country to another [12], this lack of definition reduces the possibility of generalizing the results to other contexts. A clear definition of what constitutes a nursing home in Germany would have allowed readers to draw parallels with their own contexts.

The three points raised call into question methodological choices that limit the transferability of results to other contexts. We hope that our comments and questions will stimulate constructive discussion for investigators planning to conduct a scoping review or who are interested in long-term care, as well as for stakeholders.

## Data Availability

Not applicable.

## References

[1] Skudlik S, Hirt J, Doringer T, Thalhammer R, Luftl K, Prodinger B (2023). Challenges and care strategies associated with the admission to nursing homes in Germany: a scoping review. BMC Nurs.

[2] Peters M, Godfrey C, McInerney P, Munn Z, Tricco AC, Khalil H, Aromataris E, Munn Z (2020). Chapter 11 : Scoping Reviews (2020 version). JBI Manual for Evidence Synthesis.

[3] Sussman T, Orav-Lakaski B (2020). "I Didn't Even Make My Bed": Hospital Relocations and Resident Adjustment in Long-Term Care Over Time. Gerontologist.

[4] Koppitz AL, Dreizler J, Imhof L, Altherr J, Bosshard G, Naef R (2017). Relocation experiences with unplanned admission to a nursing home: A qualitative study. Int Psychogeriatr.

[5] Campbell-Enns HJ, Campbell M, Rieger KL, Thompson GN, Doupe MB (2020). No other safe care option: Nursing home admission as a last resort strategy. Gerontologist.

[6] Weaver RH, Roberto KA, Brossoie N (2020). A scoping review: Characteristics and outcomes of residents who experience involuntary relocation. Gerontologist.

[7] Sullivan GJ, Williams C (2017). Older adult transitions into long-term care. J Gerontol Nurs.

[8] Fontaine G, Maheu-Cadotte MA, Lavallee A, Mailhot T, Lavoie P, Rouleau G (2022). Designing, planning, and conducting systematic reviews and other knowledge syntheses: Six key practical recommendations to improve feasibility and efficiency. Worldviews Evid Based Nurs.

[9] Polit D, Beck CT. Nursing research : Generating and assessing evidence for nursing practice. 11th ed. Philadelphia: Wolters Kluwer; 2021.

[10] Tricco AC, Lillie E, Zarin W, O’brien K, Colquhoun H, Kastner M (2016). A scoping review on the conduct and reporting of scoping reviews. BMC Med Res Methodol..

[11] Munn Z, Peters MD, Stern C, Tufanaru C, McArthur A, Aromataris E (2018). Systematic review or scoping review? Guidance for authors when choosing between a systematic or scoping review approach. BMC Med Res Methodol.

[12] Sanford AM, Orrell M, Tolson D, Abbatecola AM, Arai H, Bauer JM (2015). An international definition for "nursing home". J Am Med Dir Assoc.

